# Health-Related Quality of Life in Adults From 17 Family Practice Clinics in North Carolina

**Published:** 2008-12-15

**Authors:** Leigh F. Callahan, Jack Shreffler, Thelma J. Mielenz, Jay S. Kaufman, Britta Schoster, Randy Randolph, Philip Sloane, Robert DeVellis, Morris Weinberger

**Affiliations:** University of North Carolina at Chapel Hill, Thurston Arthritis Research Center; University of North Carolina at Chapel Hill; University of North Carolina at Chapel Hill; University of North Carolina at Chapel Hill; University of North Carolina at Chapel Hill; University of North Carolina at Chapel Hill; University of North Carolina at Chapel Hill; University of North Carolina at Chapel Hill; University of North Carolina at Chapel Hill, Durham Veterans Affairs Medical Center, Durham, North Carolina

## Abstract

**Introduction:**

We examined health-related quality of life (HRQOL) in white and African American patients based on their own and their community's socioeconomic status.

**Methods:**

Participants were 4,565 adults recruited from 17 family physician practices in urban and rural areas of North Carolina. Education was used as a proxy for individual socioeconomic status, and the census block-group poverty level was used as a proxy for community socioeconomic status. HRQOL measures were the 12-Item Short Form Survey Instrument, physical component summary (PCS) and mental component summary (MCS), and 3 Centers for Disease Control and Prevention HRQOL healthy days measures. Multilevel analyses examined independent associations of individual and community poverty level with HRQOL, adjusting for demographics and clustering by family practice. Analyses were stratified by race and were conducted on subgroups of arthritis and cardiovascular disease patients.

**Results:**

Among whites, all 5 HRQOL measures were significantly associated with the lowest individual socioeconomic status, and 4 HRQOL measures were associated with the lowest community socioeconomic status (MCS being the exception). Among African Americans, 4 HRQOL measures were significantly associated with the lowest individual socioeconomic status and the lowest community socioeconomic status (PCS being the exception). Arthritis and cardiovascular disease subgroup analyses showed generally analogous findings.

**Conclusion:**

Better HRQOL measures generally were associated with low levels of community poverty and high levels of education, emphasizing the need for further exploration of factors that influence health.

## Introduction

Despite the growing ability of societies to extend life and prevent disease, health disparities persist (1). As researchers and policy makers have tried to answer the question of why some societies are healthier than others, attention has focused on individual-level factors such as biology (eg, genomics), psychology (eg, coping strategies), community (eg, place of residence, work environment), and society (eg, social and economic policies).

Although a strong association is well-established between lower levels of individual socioeconomic status (SES) and poor health outcomes from many diseases throughout the developed world (1), studies increasingly suggest that community social determinants (ie, the socioeconomic environment of a person's neighborhood) may influence health (2-7), independent of the person's SES (4,8-11). Regardless of personal socioeconomic position (12,13), characteristics of the physical, social, and service environments of neighborhoods and communities may influence the lives of people who live there (14), including their health (3).

Many studies that have examined the association of both individual and community social determinants with illness and death have been conducted primarily in large urban areas (5,15). The purpose of this study was to examine the relationship between individual and community SES with health-related quality of life (HRQOL) by race in a geographically diverse sample of community-dwelling non-Hispanic whites and African Americans in a southeastern state. Study participants were recruited from a representative cohort of adult primary care patients followed in 17 practices from rural and urban areas of North Carolina. Analyses were conducted on the entire sample and on subgroups of people who self-reported the 2 most common chronic conditions and leading causes of disability, arthritis and cardiovascular disease (CVD) (16).

## Methods

### Sample

In 2001, the North Carolina Family Medicine Research Network established a practice-based cohort for primary care research (17). The practices were selected to represent the geographic and racial/ethnic diversity of North Carolina. In each of the 17 participating practices, all consecutive patients at least 18 years of age who had a scheduled appointment during a 1-month period were asked by study staff to enroll in this study (N = 7,687). Of the consecutive patients approached, 4,876 (63.4%) enrolled in our study. Participants were asked to complete a self-report questionnaire that included items on demographics, chronic conditions, health behaviors, and health status. This study focused on the 4,565 participants whose self-designated race was either white (n = 3,612) or African American (n = 953). Participants were equally distributed between urban and rural practices.

In addition to examining the whole group, we also performed analyses on subgroups of patients who reported the 2 most common chronic conditions, arthritis (white, n = 969; African American, n = 275), and CVD/hypertension (white, n = 1,271; African American, n = 469). Participants were classified as self-reporting arthritis if they reported they had osteoarthritis, rheumatoid arthritis, or fibromyalgia. They were classified as self-reporting CVD/hypertension if they reported they had heart disease, CVD, or hypertension. In the subgroup analyses, individuals appeared in both subgroup analyses if they self-reported both arthritis and CVD/hypertension. All components of the study were approved by the medical institutional review board of the University of North Carolina at Chapel Hill.

### Measures

Two self-administered instruments were used to measure HRQOL: the Medical Outcomes Study’s 12-Item Short Form Survey Instrument (SF-12v2) and the Centers for Disease Control and Prevention Health-Related Quality of Life (CDC HRQOL) healthy days measure. The SF-12v2 yields 2 summary scores: physical component summary (PCS) and mental component summary (MCS). The SF-12v2 is strongly correlated with the SF-36 and is reliable in general populations (18). Higher scores on the PCS and MCS indicate better health, and scores range from 0 to 100. The healthy days measure assesses physical and mental HRQOL (19). In this study, we used responses to the following questions as single-item indicators of HRQOL: 1) “Now thinking about your physical health, which includes physical illness and injury, for how many days during the past 30 days was your physical health not good?" 2) “Now thinking about your mental health, which includes stress, depression, and problems with emotions, for how many days during the past 30 days was your mental health not good?” and 3) “During the past 30 days, for about how many days did poor physical or mental health keep you from doing your usual activities, such as self-care, work, or recreation?” CDC HRQOL questions have been validated by statistically correlating the responses with measures from more comprehensive or established instruments such as the SF-36v2 (20). The CDC HRQOL measures have good construct validity, acceptable criteria, and known groups validity, and they have been suggested for use in surveillance and research (20).

Individual SES was defined by education level, which was categorized as low (less than high school diploma), middle (high school diploma), or high (more than high school diploma). The number of years of formal education is the socioeconomic variable most closely associated with health (21) and is commonly used in epidemiologic studies (22). Community SES was defined by the block-group poverty level (percentage of the population in households with income below the federal poverty threshold), derived by matching each participant's home address to his or her census block group, a geographic entity containing an average of approximately 1,000 residents, obtained from the 2000 US Census (23,24) with MapMarker Plus 7.2 (Empower Geographics, Des Plaines, Illinois). Only results with precise geography were used. Some studies have suggested that block-group characteristics are better indicators of the immediate SES environment than are census tract measures (4). A poverty-level category was assigned as either low, medium, or high for both racial groups, with cutpoints designed to divide each racial group into tertiles. The cutpoints for whites were poverty levels of 6.9% and 13.8%. The corresponding cutpoints for African Americans were 11.8% and 21.3%. Therefore, in absolute terms, the highest poverty category for the African Americans was more severe than the highest category for whites, and the categories established hardship in relative terms within the communities, which were largely segregated.

### Analysis

We decided to stratify by race because it is a strong correlate of education and poverty level (25). We also evaluated for effect measure modification for all social variables in all 5 of the HRQOL outcome models. For every model, a different effect was found for whites and African Americans for 1 or more of the covariates, confirming our decision to stratify.

All data were analyzed by using Stata 9.0 (StataCorp LP, College Station, Texas). Descriptive statistics were computed to describe the sample, and *t* tests and χ^2^ tests were performed to evaluate statistical differences between white and African American groups and between people with arthritis or CVD/hypertension and those without arthritis or CVD/hypertension.

Because data were collected at 17 family practice sites across the state, some correlation within sites was possible. Multilevel analyses were performed by using multiple linear regressions, specifically analysis of covariance, which examined the independent associations of education and poverty with the 5 HRQOL outcomes. These models were adjusted for age, sex, and body mass index (BMI) and for clustering by family practice site by adjusting the estimated standard errors for intra-site correlation. The adjusted mean outcomes are computed from the estimated model equation as follows: the nonreferent indicators for poverty levels (middle, high) are pov2 and pov3, respectively. The nonreferent indicators for education (middle, low) are ed2 and ed3, respectively.

HRQOL = b_0_ + b_1_ × pov2 + b_2_ × pov3 + b_3_ × ed2+ b_4_ × ed3 + b_5_ × age + b_6_ × BMI + b_7_ × sex.

In many cases, study participants in a given block group attend different family practices, obviating the possibility of building a 3-level random-effects model (block groups are not nested within practice sites). Tests of formal 2-level models (people with practice sites) did not show distinct advantage over the population average-effects model.

In the models, poverty and education appeared as indicator variables, and the most beneficial categories (high education and low poverty level) served as the references. For example, the test for significance of an outcome in the low-education group evaluated the difference from the outcome in the high-education group. Models were run for the total group and then on subgroups of people who self-reported arthritis and CVD.

## Results

Characteristics of study participants are displayed in Table 1. In the total sample, African Americans were significantly more likely to be younger, be female, and have a higher BMI, less education, and worse HRQOL (except for CDC HRQOL poor mental days) compared with whites. The block-group poverty-level tertiles were calculated separately for whites and African Americans and reflected higher poverty levels in African Americans. Patients within each of the disease-specific subgroups were significantly more likely to be older, have a higher BMI, and be less educated than the patients not in the disease subgroup. The disease-specific subgroups had significantly lower HRQOL than the whole group (with the exception of MCS for the CVD/hypertension group). The mental HRQOL showed an overall less negative effect of disease than did physical HRQOL.

Table 2 presents the adjusted means for the 5 HRQOL outcomes by education and poverty level for the white total group and the arthritis and CVD/hypertension subgroups. The highest level of education and the lowest poverty level are the referent categories. The adjusted means varied more within categories of education than within categories of poverty. For example, in the white total group, SF-12v2 PCS increased with education from 38.1 to 46.6 whereas PCS increased with decreasing poverty level from 43.8 to 46.0.

For the PCS in the total white group, groups with both the middle and low levels of education had significantly lower mean scores (poorer outcomes) than the group with the highest level of education, after adjusting for poverty level, age, BMI, sex, and family practice site. Also, for the PCS, both the middle and high poverty-level groups had significantly lower mean scores than the lowest poverty-level group, adjusting for education level and the other covariates (Table 2). In the whole population, mean SF-12v2 MCS scores were significantly poorer in the middle- and low-education groups, but we found no significant differences among the poverty-level groups.

All 3 of the CDC HRQOL healthy days measures showed significantly higher mean scores (poorer outcomes) in both the lowest education-level groups and the highest poverty-level groups, adjusting for each other and age, BMI, sex, and family practice setting (Table 2) in whites. Similar findings were identified in the subgroup analyses for participants who self-reported arthritis and those who self-reported CVD/hypertension (Table 2).

Table 3 shows the adjusted means for the 5 health status measures by education and poverty level for the African American total group and the 2 disease subgroups (arthritis and CVD/hypertension). The smaller sample size, particularly for African Americans in the arthritis subgroup, resulted in fewer findings of significant differences, but the general trend of the findings follows that seen for whites. Low individual levels of education and high poverty levels were significantly associated with poorer status in the SF-12v2, MCS, more poor physical and mental health days, and more limited activity days in the total group.

## Discussion

In this community-dwelling sample of patients living in rural and urban areas of North Carolina, both individual (education) and community (poverty level) SES measures were associated with physical and mental measures of HRQOL. A 1-day difference is considered meaningful for the CDC HRQOL healthy days scores at the individual level (26). All 3 healthy days scores had a difference of at least 1 day for both the individual and the community SES measures. The minimum clinically important difference for the SF-12v2 PCS and SF-12v2 MCS in a chronic disease has been reported as 1.26 and 2.28, respectively (27). In our study, the differences for the PCS and MCS exceeded these thresholds for both education and community poverty level. Thus, all differences reported here appear to be meaningful.

Overall, the magnitude of difference in HRQOL was greater for education compared with community poverty level. For example, for whites in any group, changes in the SF-12 PCS scores, going from low to high SES, ranged from 8.1 to 9.6 for education and from 1.7 to 2.2 for community poverty level. Although the independent effects of community socioeconomic context may be relatively small, the overall importance of community socioeconomic context to individual health may be more substantial, both because it affects all people in a community and because community context shapes the person's sense of control and all individual-level variables.

The association between lower levels of individual SES and poorer health outcomes has been documented repeatedly in various parts of the developed world (28-32). Individual-level SES can be examined according to a number of variables, including formal education level, which was used in this study, income, occupation, home ownership, race, and marital status. Formal education level is, in part, a marker for behavioral variables (22,33,34), such as self-management, problem-solving abilities, efficiency in use of medical services, capacity to cope with stress, social skills, psychological status, and economic skills, which singly or together enable people to more effectively prevent, overcome, or cope with adversity (35).

Although the body of public health research relating community factors to patterns of health and disease is well-established (36), the underlying hypothesis of this prior work proposes that factors operating at the level of the communities may affect the health outcomes of individuals. Studies in recent years have suggested that area-level or community-level variables may provide information that is not captured by individual-level variables (2,37,38). In our study, using census-based block-group poverty levels as a community-level SES indicator, we found that the community indicator was significantly associated with HRQOL after controlling for the individual indicator.

Our study has several limitations. Our data are all self-reported, and we had only education level as our measure of individual SES. Although we lacked data on income and occupation as individual SES measures, many studies have shown education to be a strong marker of individual SES. The effects of community socioeconomic level on health may be underestimated in our study because we used a crude measure of community SES, block-group poverty level from the census. Future research should begin to include information about self-defined communities or at least purposefully delineate community boundaries to more closely match theoretical constructs.

The US health care research agenda places priority on reducing disparities in health outcomes among people from different socioeconomic and ethnic groups by examining the mechanisms believed to affect health. Now, more than ever, the interest in a more explicit investigation of the complex issues about health disparities is increasing (39). However, the statement in *Healthy People 2010* recognizing that communities, states, and national organizations will need to take a multidisciplinary approach to achieving health equity is often overlooked (40). The development and communication of effective actions to reduce health disparities depends on clarifying relationships between community variables (social context), individual variables (social position), and health outcomes.

## Acknowledgments

This project was supported by a grant from the National Institute of Arthritis and Musculoskeletal and Skin Diseases Multidisciplinary Clinical Research Center, Rheumatic Diseases: P60-AR49465-01.

We thank Jennifer Milan Polinski and Shannon Currey for assistance in the establishment of the cohort. We thank the following participating family practices in the North Carolina Family Medicine Research Network for their assistance: Black River Health Services, Burgaw; Bladen Medical Associates, Elizabethtown; Blair Family Medicine, Wallace; Cabarrus Family Medicine, Concord; Cabarrus Family Medicine, Harrisburg; Cabarrus Family Medicine, Kannapolis; Cabarrus Family Medicine, Mt. Pleasant; Chatham Primary Care, Siler City; CMC-Biddle Point, Charlotte; CMC-North Park, Charlotte; Community Family Practice, Asheville; Cornerstone Medical Center, Burlington; Crissman Family Practice, Graham; Dayspring Family Medicine, Eden; Family Practice of Summerfield, Summerfield; Goldsboro Family Physicians, Goldsboro; Henderson Family Medicine Clinic, Henderson; Lumber River Family Practice, Lumberton; Moncure Community Health Center-Piedmont Family Health Services, Moncure; Orange Family Medical Group, Hillsborough; Person Family Medical Center, Roxboro; Pittsboro Family Medicine, Pittsboro; Prospect Hill Community Health Center, Prospect Hill; Robbins Family Practice, Robbins; and Village Family Medicine, Chapel Hill.

## Figures and Tables

**Table 1:** Baseline Characteristics of Study Participants, Stratified by Race, Overall and for Subgroups with Arthritis and Cardiovascular Disease, North Carolina, 2001

Characteristic^a^	White			African American		

	All (N = 3,612)	Arthritis Subgroup (n = 969)	CVD/HTN Subgroup (n = 1,271)	All (N = 953)	Arthritis Subgroup (n = 275)	CVD/HTN Subgroup (n = 469)
Age, y (SD)	47.8 (16.8)^b^	58.2 (14.9)^c^	58.1 (14.8)^c^	45.9 (16.5)^b^	56.7(14.6)^c^	53.5 (14.7)^c^
BMI, kg/m^2^ (SD)	28.7 (6.8)^b^	29.7 (7.1)^c^	30.4 (7)^c^	31.7 (8.5)^b^	33.0 (8.9)^c^	33.1 (8.7)^c^
Men, %	30.8^b^	27.2^c^	35.8^c^	24.9^b^	21.8	29.7^c^
**Education^d^, %**						
High	53.6^b^	44.3^e^	42.6^e^	39.4^b^	26.9^e^	28.4^e^
Middle	29.9	29.5	32.4	31.5	24.6	29.1
Low	16.5	26.2	25.0	29.1	48.5	42.5
**Poverty level^f^, %**						
Low	33.3	29.8^e^	29.7^e^	33.1	34.3	34.4
Middle	33.4	33.6	36.2	33.5	34.3	31.0
High	33.3	36.6	34.2	33.4	31.4	34.6
**SF-12v2 score^g ^(SD)**						
PCS	45.0 (12.3)^b^	35.6 (12.1)^c^	39.3 (12.8)^c^	42.4 (12.0)^b^	34.6 (11.7)^c^	39.1 (11.7)^c^
MCS	47.7 (11.8)^b^	46.5 (12.8)^c^	47.7 (12)	46.4 (11.7)^b^	44.2 (12.5)^c^	45.7 (11.7)
**CDC HRQOL days^h ^(SD)**						
Physical	8.1 (10.4)^b^	13.7 (12.0)^c^	11 (11.8)^c^	9.1 (10.3)^b^	14.8 (11.3)^c^	11.5 (10.9)^c^
Mental	6.9 (9.8)	8.6 (10.7)^c^	7.1 (10)	7.5 (9.8)	9.6 (10.9)^c^	8.5 (10.4)^c^
Limited activity	5.4 (9.2)^b^	9.3 (11.5)^c^	7 (10.4)^c^	6.5 (9.7)^b^	10.7 (11.7)^c^	8 (10.7)^c^

Abbreviations: CVD, cardiovascular disease; HTN, hypertension; BMI, body mass index; SF-12v2 PCS, Medical Outcomes Study's 12-Item Short Form Survey Physical Component Score; SF-12v2 MCS, Medical Outcomes Study's 12-Item Short Form Survey Mental Component Score; CDC HRQOL, Centers for Disease Control and Prevention Health Related Quality of Life.

a When percentage is not shown, mean value is indicated. Mean values for people without arthritis or CVD/HTN are not depicted in the table.

b Indicates significance at *P* < .05 for comparisons between the total white group and the total African American group.

c Indicates significance at *P* < .05 using *t* tests for comparisons between people with arthritis and those without arthritis and comparisons between people with CVD/HTN and those without CVD/HTN.

d Low is less than a high school diploma, middle is a high school diploma, and high is more than a high school diploma. High education level is the reference category.

e Indicates significance at *P* < .05 using overall Pearson χ^2^ for comparisons between people with arthritis and those without arthritis and comparisons between people with CVD/HTN and those without CVD/HTN.

f Block-group poverty level (percentage of the population in households with income below the poverty level) in tertiles that are race-specific with cut points: whites, 6.9% and 13.8%; African Americans, 11.8% and 21.3%.

g The SF-12v2 yields 2 summary scores, PCS and MCS. Higher scores on the PCS and MCS indicate better health, and scores range from 0 to 100.

h CDC HRQOL days indicate the number of days in the last 30 days that respondents suffered poor physical or mental health, or had limited activities because of poor mental or physical health.

**Table 2:** Adjusted^a^ Means and Standard Errors for Health-Related Quality of Life Measures by Education^b^ and Poverty Level^c^ and by Disease Subgroups for Whites, North Carolina, 2001

Health Status Measure		Mean (SE)		

		All (N = 2,800)	Arthritis Subgroup (N = 767)	CVD/HTN Subgroup (N = 1,002)
**SF-12v2 PCS score^d^**				
Education level	High	46.6 (0.4)	38.1 (0.8)	42.0 (0.4)
	Middle	44.6 (0.7)^e^	35.5 (1.1)^e^	40.1 (1.1)
	Low	38.1 (0.7)^e^	31.0 (0.9)^e^	32.4 (1.0)^e^
Poverty level	Low	46.0 (0.5)	36.9 (1.1)	40.3 (0.8)
	Middle	44.5 (0.6)^e^	35.2 (1.0)	39.0 (0.8)
	High	43.8 (0.6)^e^	35.0 (0.6)^e^	38.6 (0.7)
**SF-12v2 MCS score^d^**				
Education level	High	49.1 (0.4)	49.2 (0.7)	49.8 (0.6)
	Middle	47.3 (0.4)^e^	46.5 (0.9)^e^	48.3 (0.6)
	Low	44.2 (0.8)^e^	43.0 (0.6)^e^	44.0 (0.9)^e^
Poverty level	Low	48.4 (0.5)	48.0 (1.0)	48.6 (0.6)
	Middle	47.9 (0.6)	46.5 (1.0)	48.5 (0.9)
	High	47.3 (0.4)	46.5 (0.6)	46.9 (0.6)^e^
**CDC HRQOL poor physical days^f^**				
Education level	High	6.5 (0.3)	10.6 (0.7)	8.7 (0.6)
	Middle	8.6 (0.5)^e^	14.0 (0.9)^e^	10.1 (0.9)
	Low	13.3 (0.7)^e^	17.9 (1.1)^e^	16.3 (0.8)^e^
Poverty level	Low	7.5 (0.3)	12.7 (0.9)	10.1 (0.5)
	Middle	8.3 (0.5)	13.8 (1.1)	10.9 (0.9)
	High	8.6 (0.3)^e^	13.6 (0.5)	11.6 (0.6)
**CDC HRQOL poor mental days^f^**				
Education level	High	5.8 (0.2)	6.3 (0.6)	5.7 (0.5)
	Middle	7.3 (0.5)^e^	9.0 (0.9)^e^	6.6 (0.8)
	Low	9.8 (0.7)^e^	11.2 (0.8)^e^	9.8 (0.9)^e^
Poverty level	Low	6.0 (0.3)	6.8 (0.7)	5.6 (0.5)
	Middle	7.1 (0.5)^e^	9.1 (0.8)^e^	7.3 (0.7)^e^
	High	7.4 (0.7)^e^	8.7 (0.5)^e^	7.9 (0.4)^e^
**CDC HRQOL limited activity days^f^**				
Education level	High	4.2 (0.2)	6.8 (0.6)	5.1 (0.5)
	Middle	5.4 (0.3)^e^	8.9 (0.8)^e^	6.2 (0.6)
	Low	10.0 (0.8)^e^	13.5 (1.1)^e^	11.5 (0.9)^e^
Poverty level	Low	4.6 (0.3)	7.8 (0.6)	5.8 (0.6)
	Middle	5.6 (0.4)^e^	9.5 (0.8)	7.0 (0.7)
	High	5.9 (0.4)^e^	9.6 (0.7)^e^	7.8 (0.6)^e^

Abbreviations: CVD, cardiovascular disease; HTN, hypertension; SF-12v2 PCS, Medical Outcomes Study's 12-Item Short Form Survey Physical Component Summary; SF-12v2 MCS, Medical Outcomes Study's 12-Item Short Form Survey Mental Component Summary; CDC HRQOL, Centers for Disease Control and Prevention Health Related Quality of Life.

a Based on a multiple linear regression model adjusted for age, body mass index, sex, and family practice site clustering.

b Education categories are defined as low, less than a high school diploma; middle, a high school diploma; and high, more than a high school diploma.

c Block-group poverty level (percentage of the population in households with income below the poverty level) in tertiles that are race-specific with cut points: whites, 6.9% and 13.8%; African Americans, 11.8% and 21.3%.

d The SF-12v2 yields 2 summary scores, PCS and MCS. Higher scores on the PCS and MCS indicate better health, and scores range from 0 to 100.

e Indicates significance at *P* < .05, indicating that adjusted mean of category is different from the mean of reference category. Referent categories are high education and low poverty level.

f CDC HRQOL days indicate the number of days in the last 30 days that respondents suffered poor physical or mental health, or had limited activities because of poor mental or physical health.

**Table 3:** Adjusted^a^ Means and Standard Errors for Health-Related Quality of Life Measures by Education^b^ and Poverty Level^c^ and by Disease Subgroups for African Americans, North Carolina, 2001

Health Status Measure		Mean (SE)		

		All (N = 618)	Arthritis Subgroup (N = 185)	CVD/HTN Subgroup (N = 310)
**SF-12v2 PCS score^d^**				
Educationl evel	High	44.2 (0.9)	36.2 (2.0)	40.3 (0.9)
	Middle	42.2 (0.6)^e^	36.6 (1.3)	40.4 (1.1)
	Low	40.3 (1.3)	34.2 (2.1)	37.2 (1.4)
Poverty level	Low	43.4 (0.6)	36.3 (1.1)	39.9 (0.7)
	Middle	41.9 (0.8)	33.1 (2.3)	39.1 (1.1)
	High	42.3 (1.0)	37.1 (1.2)	38.3 (0.9)
**SF-12v2 MCS score^d^**				
Education level	High	48.1 (0.6)	46.3 (1.5)	48.8 (0.9)
	Middle	46.5 (0.8)	45.3 (1.3)	46.1 (1.0)^e^
	Low	44.0 (1.0)^e^	42.5 (1.9)	43.5 (0.8)^e^
Poverty level	Low	47.6 (0.7)	45.6 (0.8)	47.0 (0.6)
	Middle	46.3 (1.0)	44.2 (1.9)	45.7 (1.3)
	High	45.5 (0.8)^e^	43.2 (1.5)	45.1 (1.0)
**CDC HRQOL poor physical days^f^**				
Education level	High	7.4 (0.9)	13.1 (1.4)	9.6 (1.0)
	Middle	8.3 (0.5)	12.4 (1.1)	9.9 (1.1)
	Low	11.0 (1.0)^e^	16.0 (1.4)	13.1 (1.2)^e^
Povertyl evel	Low	7.4 (0.7)	12.4 (1.9)	9.3 (1.0)
	Middle	9.5 (0.6)^e^	16.3 (1.8)	12.2 (1.2)
	High	9.3 (0.5)^e^	14.1 (1.2)	12.0 (0.8)^e^
**CDC HRQOL poor mental days^f^**				
Education level	High	5.8 (0.5)	8.0 (0.7)	6.7 (0.9)
	Middle	7.2 (0.5)^e^	7.9 (1.0)	7.8 (0.8)
	Low	9.3 (0.9)^e^	10.6 (0.7)^e^	9.1 (0.6)
Poverty level	Low	5.5 (0.4)	7.4 (1.1)	5.8 (0.8)
	Middle	7.7 (0.7)^e^	9.7 (1.1)	9.1 (1.0)
	High	8.4 (0.5)^e^	10.6 (1.0)^e^	9.2 (0.7)^e^
**CDC HRQOL limited activity days^f^**				
Education level	High	4.9 (0.6)	8.6 (1.3)	5.9 (0.9)
	Middle	6.4 (0.7)^e^	8.3 (1.2)	7.1 (1.2)
	Low	8.8 (0.9)^e^	13.1 (1.5)^e^	9.9 (1.1)^e^
Poverty level	Low	5.3 (0.6)	10.2 (2.0)	6.5 (1.0)
	Middle	6.9 (0.7)	10.8 (1.0)	8.4 (1.4)
	High	7.1 (0.5)^e^	10.8 (1.1)	8.9 (0.7)^e^

Abbreviations: CVD, cardiovascular disease; HTN, hypertension; SF-12v2 PCS, Medical Outcomes Study's 12-Item Short Form Survey Physical Component Summary; SF-12v2 MCS, Medical Outcomes Study's 12-Item Short Form Survey Mental Component Summary; CDC HRQOL, Centers for Disease Control and Prevention Health Related Quality of Life.

a Based on a multiple linear regression model adjusted for age, body mass index, sex, and family practice site clustering.

b Low is defined as less than a high school diploma, middle, a high school diploma, and high, more than a high school diploma.

c Block-group poverty level (percentage of the population in households with income below the poverty level) in tertiles that are race-specific with cutpoints: whites, 6.9% and 13.8%; African Americans, 11.8% and 21.3%.

d The SF-12v2 yields 2 summary scores, PCS and MCS. Higher scores on the PCS and MCS indicate better health, and scores range from 0 to 100.

e Indicates significance at *P* < .05, indicating adjusted mean of class is different from the mean of reference class. Referent categories are high education and low poverty level.

f CDC HRQOL days indicate the number of days in the last 30 days that respondents suffered poor physical or mental health, or had limited activities because of poor mental or physical health.
